# Supplementary material for: Leave no child behind: Using data from 1.7 million children from 67 developing countries to measure inequality within and between groups of births and to identify left behind populations
Source: PLoS One. 2020 Oct 14;15(10):e0238847. doi: 10.1371/journal.pone.0238847 (PMC7556530; doi:10.1371/journal.pone.0238847)
Supplement: S1 Appendix — (PDF) [file pone.0238847.s001.pdf]

Appendix

A1: Tranforming the Original Wealth Index

We use the original wealth information from DHS files to construct our own wealth scores. We made the original scores more comparable across surveys, while preserving the richness of their numerical variation.

The original wealth indices were constructed using Principal Components Analysis (PCA) on household ownership information: of radios, TVs, and other domestic equipment; whether the household has electricity and clean water; type of materials used in the walls, floor and roof; and the type of toilet in the household. [36]. Scores are calculated at the household level, survey-by-survey. There are two original versions: a numeric version and a categorical wealth quintile version, based on the numerical version. Neither version is standardized across surveys and the numeric version's range varies from survey to survey.

Previous studies using these scores used the wealth quintile. Although being in a particular quintile in a particular survey is not comparable with being in the same quintile from another survey — even within the same country — quintiles can still be interpreted as the relative wealth or socioeconomic household rank in each survey. Thus being in the poorest quintile always means to be among the 20% poorest in each survey, although poverty levels are not the same. However, these scores can be and are used in a comparative fashion within each survey as a socioeconomic gradient.

We constructed a numerical variable that has the same interpretation as the quintile, while preserving within-quintile variability in wealth. This is particularly useful to aid the estimation of mortality risk. Our solution to make the numerical scores comparable across surveys is to convert them from the original numerical version, to a cumulative density function (cdf), which is bounded by the unit interval $(0, 1)$. Although our new score is a numerical score, it has the same interpretation as quintiles in terms of relative socioeconomic rank within surveys.

However, it no longer ignores within quintile variability and thus provides us a richer source of information.

The original scores are calculated at the household level, not birth level. However, we assign scores to births, as our analysis is at the birth level. Since mothers from the poorest households generally have higher fertility than mothers from richer households, quintiles of births and of household do not match perfectly. In particular, the lowest household quintile will always have more than 20% of all births and the richest household quintile will always have less than 20% of all births.

The unit of our analysis is the birth. We use our wealth quintiles in the estimation stage. We use our wealth quintiles — at the birth level, not the household level — in our inequality analysis in tabulation, box plots and ANOVA, and in the statistical model.

A 2: Model Notation and Formulation

Let $k = 1, \ldots, 182$ index surveys, $i = 1, \ldots, N_k$ index births in survey k, $j = 1, \ldots, J$ index covariates, and $m(i) \in \{1, \ldots, M_k\}$ is the ith child's geographic location (sampling cluster) in the kth country out of M_k clusters in survey k. Let y_{ik} be a binary indicator that the ith birth in country k results in death prior to five years of age, $y_{ik} = 1$, else, $y_{ik} = 0$.

Let $\boldsymbol{X}_k$ be an $N_k \times L$ design matrix with rows x_{ik}^T containing the sex of the infant, residence (urban/rural), whether or not the mother already experienced the death of a previous child, the maternal age at birth, wealth CDF birth order, birth year, mother's education in years, and functions of these variables. The continuous covariates were included in the model using piecewise transformations. For maternal age, we use a piecewise linear spline with knots at 18, 23, and 35. For wealth CDF, we use a piecewise linear spline with knots at 0.25, 0.50, and 0.75. For maternal education, we include three terms: a binary indicator for maternal education greater than 13 years and two terms corresponding to a piecewise linear spline with knot at 5 years for maternal education less than 13 years. For birth order, we include a binary indicator that birth order equal one, a binary indicator that birth order is six or more, and a linear term for birth order between two and 6. Additionally, all two, three, and four-sway interactions were included in the model, using untransformed values for the continuous variables instead of the splines.

We model y_{ik} using a random effects logistic regression,

$$y_{ik} \sim \text{Bernoulli}(\pi_{ik})$$

$$\text{Logit}(\pi_{ik}) = \alpha_{0k} + x_{ik}^T \boldsymbol{\alpha}_k + b_{m_{(i)}},$$

where $\boldsymbol{\alpha}_{0k}$ is an intercept and $\boldsymbol{\alpha}_k$ is a vector of regression coefficients,

$$\text{Logit}(\pi_{ik}) = \log\left(\frac{\pi_{ik}}{1 - \pi_{ik}}\right)$$

and in the kth survey

$$b_m|\tau_k^2 \sim \mathrm{N}\left(Fig0, \tau_k^2\right)$$

is a random effect for location m.

Prior Specification

For all k surveys, the variance parameters τ_k^2 are given Inverse-Gamma$(3, 1.5)$ priors, and the elements α_{jk} of $\boldsymbol{\alpha}_k$ are given normal priors,

$$\alpha_{jk} \sim \mathrm{N}\left(0, 2^{1-c_j}\right)$$

where c_j is the *order* of the interaction so that $c_j = 1$ for the intercept and main effects, $c_j = 2$ for two-way interactions, $c_j = 3$ for three-way interactions, and $c_j = 4$ for four-way interactions. These priors shrink higher order interactions terms closer to zero to avoid overfitting.

A 3: Bayesian ANOVA

Let Π be the distribution of mortality risk in a country and $\text{Var}(\Pi)$ the variance of mortality risk. $\text{Var}(\Pi)$ can be expressed as the between group variance plus the sum of the variances within each group. Let $\mathbf{X}$ be a categorical or continuous covariate. Using the law of total variance we have the decomposition

$$\text{Var}(\Pi) \quad = \quad \text{E}(\text{Var}[\Pi|\text{X}]) + \text{Var}(\text{E}[\Pi|\text{X}]) \tag{1}$$

where for categorical variables $\text{E}(\text{Var}[\Pi|\text{X}])$ is the average within group variance and $\text{Var}(\text{E}[\Pi|\text{X}])$ is the between-group variance of the group means. We fit linear regression models using OLS methods where mortality risk is the outcome and group membership is the predictor. We use R^2 to measure how much of the total variance in Π can be explained by membership in a particular socioeconomic group.

To propagate uncertainty from the estimation stage to the analysis of inequality stage we calculate an ANOVA for each MCMC sample giving a distribution of R^2. We use 1000 MCMC samples. We can also use this approach to make probabilistic statements, such as what is the probability that inequality is greater in one year than in another year.

A 4: Additional Tables

Country	Low Risk	High Risk	Difference
Albania	27.6 (27.4, 27.8)	27.2 (26.4, 28.0)	-0.4 (-1.4, 0.5)
Armenia	25.0 (24.8, 25.2)	23.8 (22.9, 24.7)	-1.2 (-2.2, -0.1)
Angola	25.5 (25.3, 25.7)	25.3 (24.5, 26.1)	-0.2 (-1.2, 0.9)
Azerbaijan	25.7 (25.5, 25.9)	25.5 (24.6, 26.4)	-0.2 (-1.3, 1.0)
Bangladesh	23.5 (23.3, 23.7)	24.1 (23.3, 24.8)	0.6 (-0.4, 1.6)
Burkina Faso	26.7 (26.5, 26.8)	25.9 (25.2, 26.6)	-0.8 (-1.6, 0.1)
Benin	26.2 (26.0, 26.3)	26.0 (25.5, 26.6)	-0.2 (-0.8, 0.5)
Bolivia	26.6 (26.4, 26.8)	28.1 (27.2, 28.9)	1.5 (0.4, 2.4)
Brazil	25.8 (25.7, 26.0)	26.1 (25.4, 26.8)	0.2 (-0.7, 1.2)
Burundi	28.2 (28.0, 28.4)	26.6 (25.7, 27.6)	-1.6 (-2.7, -0.3)
Congo Democratic Republic	26.6 (26.5, 26.8)	27.0 (26.3, 27.7)	0.4 (-0.5, 1.3)
Central African Republic	25.9 (25.7, 26.1)	24.7 (23.8, 25.7)	-1.2 (-2.4, 0.0)
Congo	26.2 (26.0, 26.4)	27.0 (26.1, 28.0)	0.8 (-0.4, 2.0)
Côte d'Ivoire	25.8 (25.6, 26.0)	26.9 (26.1, 27.7)	1.1 (0.1, 2.1)
Cameroon	25.8 (25.7, 26.0)	25.7 (25.0, 26.4)	-0.2 (-1.1, 0.7)
Colombia	25.7 (25.5, 25.9)	25.0 (24.2, 25.8)	-0.8 (-1.7, 0.3)
Dominican Republic	24.7 (24.5, 24.9)	23.3 (22.6, 24.2)	-1.3 (-2.3, -0.2)
Egypt	26.5 (26.4, 26.7)	25.8 (25.1, 26.4)	-0.7 (-1.6, 0.1)
Ethiopia	26.4 (26.2, 26.5)	25.7 (25.0, 26.4)	-0.7 (-1.5, 0.2)
Gabon	26.4 (26.2, 26.7)	26.7 (25.7, 27.7)	0.3 (-0.9, 1.6)
Ghana	27.6 (27.4, 27.9)	27.4 (26.5, 28.3)	-0.3 (-1.4, 0.9)
Guinea	26.2 (26.0, 26.5)	26.5 (25.6, 27.4)	0.3 (-0.9, 1.5)
Guatemala	26.0 (25.8, 26.2)	26.5 (25.5, 27.4)	0.5 (-0.8, 1.7)
Guyana	26.2 (25.9, 26.5)	26.8 (25.7, 27.9)	0.7 (-0.8, 2.1)
Honduras	25.7 (25.4, 25.9)	25.5 (24.6, 26.4)	-0.2 (-1.4, 1.0)
Haiti	27.5 (27.3, 27.8)	28.1 (27.1, 29.0)	0.6 (-0.6, 1.8)
India	24.6 (24.5, 24.6)	24.1 (23.7, 24.4)	-0.5 (-0.9, 0.0)
Indonesia	27.6 (27.4, 27.7)	27.2 (26.6, 27.8)	-0.4 (-1.1, 0.3)
Jordan	28.3 (28.1, 28.6)	28.7 (27.8, 29.6)	0.4 (-0.8, 1.5)
Kenya	25.9 (25.8, 26.1)	26.0 (25.4, 26.7)	0.1 (-0.6, 0.9)
Cambodia	26.5 (26.3, 26.7)	28.8 (28.1, 29.6)	2.3 (1.3, 3.3)
Kazakhstan	26.5 (26.2, 26.7)	25.5 (24.6, 26.5)	-1.0 (-2.1, 0.2)
Comoros	26.5 (26.3, 26.8)	27.1 (26.2, 28.1)	0.6 (-0.6, 1.7)
Kyrgyzstan	27.0 (26.8, 27.3)	26.0 (25.0, 27.0)	-1.0 (-2.3, 0.2)
Liberia	26.5 (26.3, 26.8)	26.2 (25.3, 27.1)	-0.3 (-1.4, 0.8)
Lesotho	26.0 (25.7, 26.2)	26.0 (25.1, 26.9)	0.0 (-1.0, 1.2)
Morocco	28.6 (28.4, 28.8)	27.3 (26.4, 28.2)	-1.3 (-2.5, -0.2)
Moldova	25.3 (25.2, 25.5)	24.6 (23.9, 25.3)	-0.8 (-1.6, 0.1)
Madagascar	26.0 (25.8, 26.2)	27.0 (26.2, 27.7)	1.0 (0.1, 1.9)
Mali	25.6 (25.5, 25.8)	25.4 (24.8, 26.1)	-0.2 (-1.0, 0.6)
Malawi	25.6 (25.4, 25.7)	26.3 (25.6, 27.0)	0.8 (-0.1, 1.6)
Mozambique	26.1 (25.9, 26.2)	24.4 (23.7, 25.0)	-1.7 (-2.6, -0.9)
Nicaragua	25.7 (25.5, 25.9)	24.8 (23.9, 25.7)	-0.9 (-2.0, 0.3)
Nigeria	26.8 (26.7, 26.9)	26.6 (26.2, 27.1)	-0.2 (-0.7, 0.4)
Niger	26.0 (25.8, 26.2)	25.4 (24.7, 26.1)	-0.6 (-1.5, 0.3)
Namibia	26.6 (26.4, 26.9)	27.1 (26.1, 28.0)	0.4 (-0.7, 1.5)
Peru	27.1 (26.9, 27.3)	26.6 (25.9, 27.4)	-0.5 (-1.4, 0.5)
Philippines	27.9 (27.7, 28.2)	28.2 (27.2, 29.2)	0.3 (-1.0, 1.6)
Pakistan	27.0 (26.8, 27.1)	26.9 (26.3, 27.6)	-0.1 (-0.9, 0.7)
Paraguay	27.3 (27.1, 27.6)	27.1 (26.0, 28.2)	-0.2 (-1.6, 1.1)
Rwanda	27.7 (27.5, 27.9)	27.9 (27.1, 28.7)	0.2 (-0.8, 1.3)
Sierra Leone	26.3 (26.1, 26.5)	26.9 (26.2, 27.6)	0.6 (-0.3, 1.5)
Senegal	26.8 (26.5, 27.0)	26.8 (25.9, 27.7)	0.1 (-1.0, 1.2)
Sao Tome and Principe	26.0 (25.8, 26.3)	26.6 (25.5, 27.7)	0.6 (-0.8, 2.0)
Eswatini	25.8 (25.5, 26.1)	27.0 (25.9, 28.0)	1.2 (-0.2, 2.5)
Chad	25.6 (25.5, 25.7)	25.8 (25.2, 26.3)	0.2 (-0.5, 0.9)
Togo	27.5 (27.3, 27.7)	27.7 (26.9, 28.6)	0.2 (-1.0, 1.3)
Timor-Leste	29.2 (29.0, 29.4)	27.4 (26.6, 28.1)	-1.8 (-2.7, -0.9)
Turkey	25.9 (25.7, 26.1)	25.5 (24.7, 26.3)	-0.4 (-1.3, 0.6)
Tanzania	27.0 (26.7, 27.2)	27.7 (26.8, 28.7)	0.8 (-0.5, 2.0)
Ukraine	24.7 (24.4, 24.9)	24.9 (24.1, 25.8)	0.3 (-0.8, 1.5)
Uganda	26.0 (25.8, 26.2)	26.0 (25.2, 26.9)	0.0 (-1.1, 1.1)
Uzbekistan	26.5 (26.2, 26.7)	25.5 (24.6, 26.4)	-1.0 (-2.0, 0.2)
Vietnam	26.8 (26.5, 27.0)	26.7 (25.7, 27.7)	0.0 (-1.2, 1.2)
South Africa	26.5 (26.3, 26.8)	26.7 (25.8, 27.6)	0.1 (-1.0, 1.3)
Zambia	25.9 (25.8, 26.1)	27.4 (26.6, 28.0)	1.4 (0.5, 2.3)
Zimbabwe	25.6 (25.4, 25.8)	25.7 (24.9, 26.5)	0.1 (-0.9, 1.1)

Table 1 — Comparison of maternal age (in years) between high risk and non-high risk births. Latest survey for each country.

Country	Low Risk	High Risk	Difference
Albania	9.95 (9.85, 10.03)	9.11 (8.77, 9.51)	-0.84 (-1.27, -0.35)
Armenia	11.75 (11.63, 11.86)	11.22 (10.77, 11.70)	-0.53 (-1.10, 0.08)
Angola	4.11 (4.02, 4.19)	2.4 (2.06, 2.73)	-1.70 (-2.1, -1.29)
Azerbaijan	10.64 (10.56, 10.73)	10.19 (9.82, 10.53)	-0.46 (-0.91, -0.03)
Bangladesh	5.29 (5.18, 5.40)	2.84 (2.41, 3.32)	-2.45 (-2.99, -1.84)
Burkina Faso	0.82 (0.79, 0.84)	0.25 (0.17, 0.35)	-0.56 (-0.67, -0.44)
Benin	1.24 (1.19, 1.27)	0.74 (0.60, 0.91)	-0.49 (-0.67, -0.28)
Bolivia	7.09 (6.98, 7.20)	3.68 (3.25, 4.12)	-3.41 (-3.93, -2.86)
Brazil	5.63 (5.54, 5.72)	2.72 (2.36, 3.1)	-2.91 (-3.36, -2.44)
Burundi	2.70 (2.62, 2.78)	1.76 (1.45, 2.09)	-0.94 (-1.34, -0.53)
Congo Democratic Republic	4.86 (4.78, 4.94)	3.28 (2.98, 3.6)	-1.59 (-1.99, -1.18)
Central African Republic	2.02 (1.95, 2.09)	1.13 (0.87, 1.4)	-0.89 (-1.21, -0.55)
Congo	6.20 (6.09, 6.31)	4.82 (4.4, 5.26)	-1.39 (-1.92, -0.84)
Côte d'Ivoire	1.73 (1.68, 1.78)	0.79 (0.59, 1.01)	-0.94 (-1.18, -0.66)
Cameroon	5.10 (5.02, 5.19)	2.23 (1.9, 2.57)	-2.87 (-3.29, -2.46)
Colombia	7.46 (7.34, 7.57)	5.40 (4.94, 5.86)	-2.08 (-2.65, -1.49)
Dominican Republic	8.76 (8.57, 8.95)	7.03 (6.29, 7.79)	-1.73 (-2.65, -0.77)
Egypt	8.54 (8.37, 8.72)	5.81 (5.07, 6.49)	-2.74 (-3.69, -1.87)
Ethiopia	1.31 (1.28, 1.34)	0.47 (0.36, 0.59)	-0.84 (-0.99, -0.69)
Gabon	6.47 (6.36, 6.57)	5.62 (5.21, 6.05)	-0.85 (-1.37, -0.32)
Ghana	4.81 (4.67, 4.95)	2.56 (2.03, 3.14)	-2.25 (-2.93, -1.54)
Guinea	1.02 (0.98, 1.06)	0.47 (0.33, 0.65)	-0.55 (-0.73, -0.33)
Guatemala	4.62 (4.51, 4.72)	2.41 (2.01, 2.85)	-2.20 (-2.71, -1.63)
Guyana	7.86 (7.71, 8.02)	7.24 (6.60, 7.81)	-0.64 (-1.42, 0.04)
Honduras	5.50 (5.38, 5.64)	4.80 (4.26, 5.32)	-0.70 (-1.38, -0.05)
Haiti	3.88 (3.79, 3.98)	1.84 (1.46, 2.23)	-2.03 (-2.51, -1.54)
India	4.78 (4.74, 4.82)	1.19 (1.03, 1.35)	-3.60 (-3.79, -3.39)
Indonesia	8.99 (8.91, 9.08)	5.74 (5.40, 6.08)	-3.25 (-3.66, -2.82)
Jordan	10.86 (10.73, 11)	9.62 (9.07, 10.14)	-1.25 (-1.97, -0.61)
Kenya	6.11 (6.02, 6.19)	5.75 (5.40, 6.10)	-0.36 (-0.79, 0.08)
Cambodia	4.61 (4.51, 4.69)	2.26 (1.93, 2.63)	-2.35 (-2.76, -1.89)
Kazakhstan	11.11 (11.02, 11.19)	10.61 (10.29, 10.96)	-0.50 (-0.92, -0.06)
Comoros	3.38 (3.24, 3.53)	2.57 (2.00, 3.15)	-0.80 (-1.50, -0.07)
Kyrgyzstan	12.04 (11.93, 12.15)	11.72 (11.30, 12.17)	-0.32 (-0.85, 0.26)
Liberia	2.43 (2.34, 2.51)	1.90 (1.57, 2.24)	-0.53 (-0.95, -0.11)
Lesotho	7.23 (7.13, 7.32)	7.04 (6.66, 7.45)	-0.19 (-0.67, 0.30)
Morocco	2.10 (2.00, 2.18)	0.87 (0.56, 1.27)	-1.23 (-1.62, -0.71)
Moldova	11.36 (11.26, 11.46)	11.05 (10.68, 11.46)	-0.33 (-0.80, 0.14)
Madagascar	3.55 (3.49, 3.6)	2.04 (1.81, 2.27)	-1.50 (-1.78, -1.19)
Mali	1.04 (1.00, 1.07)	0.48 (0.35, 0.61)	-0.56 (-0.71, -0.39)
Malawi	5.35 (5.27, 5.44)	3.88 (3.55, 4.22)	-1.48 (-1.89, -1.04)
Mozambique	2.98 (2.91, 3.05)	2.25 (1.99, 2.52)	-0.73 (-1.08, -0.40)
Nicaragua	4.56 (4.45, 4.67)	2.43 (1.97, 2.87)	-2.13 (-2.73, -1.58)
Nigeria	4.88 (4.83, 4.94)	1.19 (0.98, 1.39)	-3.70 (-3.95, -3.44)
Niger	0.82 (0.79, 0.84)	0.39 (0.28, 0.50)	-0.43 (-0.56, -0.28)
Namibia	7.77 (7.63, 7.91)	7.15 (6.58, 7.72)	-0.63 (-1.34, 0.09)
Peru	8.18 (8.08, 8.27)	5.50 (5.12, 5.89)	-2.68 (-3.15, -2.19)
Philippines	9.35 (9.22, 9.47)	6.67 (6.20, 7.21)	-2.67 (-3.25, -2.02)
Pakistan	3.22 (3.16, 3.29)	1.05 (0.81, 1.32)	-2.18 (-2.48, -1.84)
Paraguay	5.33 (5.24, 5.41)	4.06 (3.72, 4.41)	-1.27 (-1.70, -0.83)
Rwanda	4.33 (4.23, 4.43)	3.48 (3.11, 3.88)	-0.85 (-1.32, -0.33)
Sierra Leone	1.55 (1.50, 1.60)	0.96 (0.75, 1.16)	-0.59 (-0.84, -0.34)
Senegal	1.44 (1.40, 1.48)	0.51 (0.35, 0.68)	-0.93 (-1.12, -0.71)
Sao Tome and Principe	4.53 (4.44, 4.61)	3.52 (3.19, 3.87)	-1.01 (-1.42, -0.55)
Eswatini	7.76 (7.60, 7.92)	6.29 (5.65, 6.94)	-1.48 (-2.26, -0.65)
Chad	1.03 (0.98, 1.07)	1.43 (1.25, 1.62)	0.40 (0.17, 0.63)
Togo	2.57 (2.48, 2.64)	1.67 (1.36, 2.00)	-0.90 (-1.28, -0.48)
Timor-Leste	5.26 (5.13, 5.39)	3.60 (3.10, 4.12)	-1.66 (-2.29, -0.99)
Turkey	4.66 (4.52, 4.78)	2.36 (1.88, 2.92)	-2.30 (-2.91, -1.59)
Tanzania	5.19 (5.08, 5.30)	4.43 (4.00, 4.88)	-0.77 (-1.33, -0.22)
Ukraine	13.29 (13.11, 13.46)	14.13 (13.47, 14.85)	0.87 (0.03, 1.82)
Uganda	4.45 (4.34, 4.56)	2.95 (2.54, 3.40)	-1.50 (-2.02, -0.96)
Uzbekistan	10.58 (10.51, 10.64)	10.58 (10.34, 10.86)	0.00 (-0.29, 0.34)
Vietnam	6.99 (6.82, 7.15)	6.07 (5.43, 6.74)	-0.90 (-1.75, -0.04)
South Africa	7.76 (7.61, 7.90)	5.80 (5.23, 6.38)	-1.95 (-2.68, -1.19)
Zambia	5.76 (5.66, 5.85)	4.58 (4.20, 4.97)	-1.17 (-1.64, -0.68)
Zimbabwe	9.30 (9.21, 9.39)	7.69 (7.33, 8.06)	-1.61 (-2.05, -1.16)

Table 2 — Comparison of maternal education (in years) between non-high risk and high risk births. Latest survey for each country.

Country	Low Risk	High Risk	Odds Ratio
Albania	53.0% (51.0%, 55.1%)	57.7% (49.3%, 65.6%)	1.2 (0.8, 1.8)
Armenia	52.3% (49.3%, 55.3%)	60.2% (48.2%, 72.2%)	1.5 (0.8, 2.7)
Angola	51.6% (50.1%, 53.2%)	54.6% (48.2%, 60.8%)	1.1 (0.8, 1.5)
Azerbaijan	53.0% (50.8%, 55.2%)	56.7% (47.6%, 65.5%)	1.2 (0.7, 1.8)
Bangladesh	51.1% (49.5%, 52.9%)	52.2% (45.2%, 58.6%)	1.1 (0.7, 1.4)
Burkina Faso	50.2% (48.9%, 51.5%)	56.8% (51.4%, 61.8%)	1.3 (1.0, 1.7)
Benin	50.7% (49.7%, 51.8%)	55.2% (51.1%, 59.5%)	1.2 (1.0, 1.5)
Bolivia	50.1% (48.5%, 51.6%)	54.3% (48.2%, 60.6%)	1.2 (0.9, 1.6)
Brazil	49.3% (47.7%, 50.9%)	57.3% (51.1%, 63.6%)	1.4 (1.0, 1.9)
Burundi	47.4% (45.7%, 49.3%)	58.8% (51.5%, 65.8%)	1.6 (1.1, 2.3)
Congo Democratic Republic	48.7% (47.6%, 49.9%)	54.2% (49.1%, 58.7%)	1.3 (1.0, 1.6)
Central African Republic	49.0% (47.0%, 50.9%)	60.0% (52.5%, 68.1%)	1.6 (1.1, 2.4)
Congo	50.0% (48.2%, 51.7%)	53.5% (46.6%, 60.6%)	1.2 (0.8, 1.7)
Côte d'Ivoire	49.2% (47.9%, 50.7%)	60.6% (54.7%, 65.9%)	1.6 (1.2, 2.1)
Cameroon	48.5% (47.2%, 49.8%)	59.4% (54.3%, 64.6%)	1.6 (1.2, 2.0)
Colombia	49.1% (47.6%, 50.5%)	60.6% (54.8%, 66.5%)	1.6 (1.2, 2.2)
Dominican Republic	51.6% (49.5%, 53.9%)	51.1% (41.9%, 59.5%)	1.0 (0.6, 1.5)
Egypt	51.5% (49.9%, 53.1%)	51.1% (44.7%, 57.2%)	1.0 (0.7, 1.3)
Ethiopia	48.4% (47.0%, 49.7%)	60.8% (55.7%, 66.2%)	1.7 (1.3, 2.2)
Gabon	47.6% (45.7%, 49.3%)	54.7% (47.7%, 62.0%)	1.4 (0.9, 1.9)
Ghana	48.3% (46.4%, 50.0%)	61.0% (54.3%, 68.7%)	1.7 (1.2, 2.5)
Guinea	49.3% (47.7%, 50.9%)	54.8% (48.4%, 61.2%)	1.3 (0.9, 1.7)
Guatemala	49.5% (47.7%, 51.3%)	51.2% (44.0%, 58.6%)	1.1 (0.7, 1.6)
Guyana	52.0% (49.7%, 54.4%)	43.7% (34.1%, 53.1%)	0.7 (0.4, 1.1)
Honduras	50.7% (48.7%, 52.6%)	52.1% (44.5%, 59.9%)	1.1 (0.7, 1.6)
Haiti	49.2% (47.4%, 50.8%)	60.6% (54.0%, 67.7%)	1.6 (1.1, 2.3)
India	52.1% (51.3%, 52.8%)	49.9% (47.0%, 53.0%)	0.9 (0.8, 1.1)
Indonesia	49.2% (48.2%, 50.3%)	62.4% (57.9%, 66.5%)	1.7 (1.4, 2.1)
Jordan	48.0% (46.2%, 50.0%)	64.4% (56.5%, 71.6%)	2.0 (1.3, 2.9)
Kenya	50.4% (49.2%, 51.6%)	53.2% (48.3%, 57.8%)	1.1 (0.9, 1.4)
Cambodia	49.0% (47.4%, 50.6%)	60.6% (54.2%, 67.0%)	1.6 (1.2, 2.3)
Kazakhstan	51.2% (48.9%, 53.7%)	59.4% (49.4%, 68.4%)	1.4 (0.8, 2.3)
Comoros	52.3% (50.3%, 54.3%)	44.7% (36.8%, 52.9%)	0.8 (0.5, 1.1)
Kyrgyzstan	51.5% (48.9%, 54.1%)	54.3% (44.0%, 64.9%)	1.2 (0.7, 1.9)
Liberia	49.6% (48.1%, 51.2%)	56.7% (50.4%, 62.8%)	1.3 (1.0, 1.8)
Lesotho	48.2% (46.4%, 50.4%)	60.6% (52.0%, 68.1%)	1.7 (1.1, 2.5)
Morocco	49.4% (47.7%, 51.2%)	54.6% (47.3%, 61.3%)	1.3 (0.9, 1.7)
Moldova	48.7% (46.6%, 50.7%)	64.5% (56.8%, 73.0%)	2.0 (1.3, 3.1)
Madagascar	50.3% (49.0%, 51.6%)	55.8% (50.6%, 60.8%)	1.3 (1.0, 1.6)
Mali	48.6% (47.3%, 49.8%)	61.8% (57.0%, 66.8%)	1.7 (1.3, 2.2)
Malawi	47.1% (45.8%, 48.3%)	62.3% (57.4%, 67.4%)	1.9 (1.4, 2.4)
Mozambique	48.2% (46.9%, 49.6%)	54.7% (49.0%, 59.9%)	1.3 (1.0, 1.7)
Nicaragua	48.5% (46.8%, 50.4%)	62.1% (54.7%, 69.0%)	1.8 (1.2, 2.5)
Nigeria	50.4% (49.7%, 51.2%)	53.8% (50.7%, 56.8%)	1.1 (1.0, 1.3)
Niger	49.0% (47.7%, 50.2%)	61.6% (56.6%, 66.7%)	1.7 (1.3, 2.2)
Namibia	48.0% (46.0%, 50.0%)	61.5% (53.5%, 69.6%)	1.8 (1.1, 2.7)
Peru	48.1% (46.8%, 49.4%)	60.6% (55.3%, 65.8%)	1.7 (1.3, 2.2)
Philippines	49.1% (47.3%, 50.9%)	57.9% (50.7%, 65.2%)	1.5 (1.0, 2.1)
Pakistan	52.8% (51.5%, 54.3%)	51.6% (45.9%, 56.9%)	1.0 (0.7, 1.2)
Paraguay	50.9% (48.8%, 53.0%)	53.6% (45.2%, 61.9%)	1.1 (0.7, 1.7)
Rwanda	48.6% (46.8%, 50.5%)	55.1% (47.9%, 62.5%)	1.3 (0.9, 1.9)
Sierra Leone	50.6% (49.4%, 51.8%)	55.6% (50.7%, 60.4%)	1.2 (1.0, 1.6)
Senegal	50.5% (49.0%, 52.2%)	55.4% (48.7%, 61.6%)	1.2 (0.9, 1.7)
Sao Tome and Principe	49.1% (46.9%, 51.4%)	48.6% (39.5%, 57.6%)	1.0 (0.6, 1.5)
Eswatini	50.8% (48.6%, 52.9%)	56.4% (48.0%, 65.1%)	1.3 (0.8, 2.0)
Chad	50.8% (49.8%, 51.9%)	55.4% (51.3%, 59.3%)	1.2 (1.0, 1.5)
Togo	49.8% (48.2%, 51.4%)	55.0% (48.5%, 61.7%)	1.3 (0.9, 1.7)
Timor-Leste	50.8% (49.2%, 52.3%)	54.7% (48.6%, 60.8%)	1.2 (0.9, 1.6)
Turkey	50.7% (48.9%, 52.5%)	49.8% (42.7%, 57.2%)	1.0 (0.7, 1.4)
Tanzania	49.3% (47.5%, 51.0%)	52.1% (45.1%, 59.2%)	1.1 (0.8, 1.6)
Ukraine	53.7% (50.7%, 56.5%)	49.8% (38.8%, 61.8%)	0.9 (0.5, 1.6)
Uganda	47.6% (45.9%, 49.3%)	59.8% (53.0%, 66.5%)	1.7 (1.2, 2.3)
Uzbekistan	49.5% (47.1%, 51.9%)	58.1% (48.5%, 67.6%)	1.5 (0.9, 2.3)
Vietnam	47.2% (44.9%, 49.5%)	66.9% (57.7%, 76.1%)	2.4 (1.4, 3.9)
South Africa	48.1% (46.3%, 50.1%)	60.2% (52.6%, 67.4%)	1.7 (1.1, 2.4)
Zambia	48.8% (47.4%, 50.1%)	57.5% (52.3%, 62.9%)	1.4 (1.1, 1.9)
Zimbabwe	49.4% (47.7%, 51.2%)	57.5% (50.6%, 64.4%)	1.4 (1.0, 2.0)

Table 3 — Comparison of gender (proportion female) between non-high risk and high risk births. Latest survey for each country.

Country	Low Risk	High Risk	Odds Ratio
Albania	51.9% (49.9%, 53.7%)	23.1% (15.7%, 31.0%)	0.3 (0.2, 0.5)
Armenia	64.8% (62.2%, 67.7%)	67.8% (56.4%, 78.3%)	1.2 (0.6, 2.2)
Angola	40.8% (39.4%, 42.2%)	27.7% (22.1%, 33.3%)	0.6 (0.4, 0.8)
Azerbaijan	49.0% (46.8%, 51.3%)	37.7% (28.6%, 46.5%)	0.6 (0.4, 1.0)
Bangladesh	33.9% (32.5%, 35.1%)	21.6% (16.9%, 27.1%)	0.5 (0.4, 0.8)
Burkina Faso	21.1% (20.3%, 21.9%)	15.2% (12.0%, 18.5%)	0.7 (0.5, 0.9)
Benin	36.5% (35.6%, 37.3%)	26.0% (22.5%, 29.7%)	0.6 (0.5, 0.8)
Bolivia	53.0% (51.7%, 54.4%)	31.2% (25.8%, 36.6%)	0.4 (0.3, 0.5)
Brazil	76.1% (74.7%, 77.6%)	62.4% (56.4%, 67.9%)	0.5 (0.4, 0.7)
Burundi	18.4% (17.7%, 19.1%)	7.5% (4.9%, 10.5%)	0.4 (0.2, 0.5)
Congo Democratic Republic	31.1% (30.2%, 32.1%)	20.2% (16.4%, 24.0%)	0.6 (0.4, 0.7)
Central African Republic	40.8% (39.2%, 42.4%)	27.7% (21.5%, 34.1%)	0.6 (0.4, 0.8)
Congo	24.4% (23.2%, 25.5%)	17.9% (13.5%, 22.8%)	0.7 (0.5, 1.0)
Côte d'Ivoire	31.6% (30.4%, 32.8%)	24.1% (19.3%, 29.1%)	0.7 (0.5, 0.9)
Cameroon	43.6% (42.6%, 44.5%)	15.6% (11.9%, 19.6%)	0.2 (0.2, 0.3)
Colombia	71.5% (70.0%, 73.1%)	59.9% (53.6%, 65.8%)	0.6 (0.4, 0.8)
Dominican Republic	57.8% (55.5%, 60.1%)	47.9% (38.9%, 57.1%)	0.7 (0.4, 1.1)
Egypt	46.4% (45.0%, 47.8%)	27.9% (22.2%, 33.4%)	0.5 (0.3, 0.6)
Ethiopia	18.8% (18.1%, 19.5%)	10.3% (7.6%, 13.2%)	0.5 (0.3, 0.7)
Gabon	61.9% (60.3%, 63.7%)	53.5% (46.4%, 60.2%)	0.7 (0.5, 1.0)
Ghana	40.3% (38.6%, 42.0%)	32.8% (26.3%, 39.7%)	0.7 (0.5, 1.0)
Guinea	29.3% (28.4%, 30.2%)	14.8% (11.2%, 18.2%)	0.4 (0.3, 0.6)
Guatemala	39.1% (37.6%, 40.5%)	22.5% (16.6%, 28.5%)	0.5 (0.3, 0.7)
Guyana	19.2% (17.6%, 20.8%)	21.7% (15.6%, 28.4%)	1.2 (0.7, 1.9)
Honduras	34.0% (32.1%, 35.7%)	28.8% (22.0%, 36.5%)	0.8 (0.5, 1.2)
Haiti	32.6% (31.1%, 34.1%)	34.4% (28.4%, 40.6%)	1.1 (0.8, 1.5)
India	42.0% (41.3%, 42.6%)	20.1% (17.5%, 22.6%)	0.3 (0.3, 0.4)
Indonesia	49.3% (48.2%, 50.4%)	23.9% (19.6%, 28.5%)	0.3 (0.2, 0.4)
Jordan	69.0% (67.4%, 70.6%)	72.3% (65.8%, 78.8%)	1.2 (0.8, 1.8)
Kenya	30.4% (29.4%, 31.4%)	27.8% (23.7%, 32.0%)	0.9 (0.7, 1.1)
Cambodia	29.4% (28.4%, 30.2%)	10.2% (6.9%, 14.0%)	0.3 (0.2, 0.4)
Kazakhstan	51.1% (48.6%, 53.5%)	39.2% (29.6%, 49.0%)	0.6 (0.4, 1.0)
Comoros	39.5% (37.8%, 41.0%)	19.2% (13.0%, 25.8%)	0.4 (0.2, 0.6)
Kyrgyzstan	28.0% (25.7%, 30.1%)	26.2% (17.5%, 35.1%)	0.9 (0.5, 1.6)
Liberia	33.1% (31.8%, 34.4%)	28.1% (23.1%, 33.5%)	0.8 (0.6, 1.1)
Lesotho	24.3% (22.4%, 26.0%)	31.3% (24.5%, 38.6%)	1.5 (0.9, 2.2)
Morocco	47.4% (45.9%, 49.0%)	24.9% (18.7%, 31.0%)	0.4 (0.2, 0.5)
Moldova	48.5% (46.3%, 50.4%)	38.6% (30.9%, 47.2%)	0.7 (0.4, 1.0)
Madagascar	20.2% (19.5%, 20.8%)	9.8% (7.3%, 12.6%)	0.4 (0.3, 0.6)
Mali	26.3% (25.6%, 26.9%)	10.1% (7.5%, 12.9%)	0.3 (0.2, 0.4)
Malawi	17.6% (16.9%, 18.3%)	9.8% (7.2%, 12.9%)	0.5 (0.3, 0.7)
Mozambique	32.8% (31.6%, 34.0%)	29.3% (24.6%, 34.4%)	0.9 (0.6, 1.1)
Nicaragua	46.6% (44.9%, 48.2%)	30.8% (24.2%, 37.3%)	0.5 (0.3, 0.7)
Nigeria	35.3% (34.7%, 35.8%)	13.7% (11.6%, 16.0%)	0.3 (0.2, 0.4)
Niger	23.8% (23.2%, 24.3%)	5.8% (3.9%, 8.0%)	0.2 (0.1, 0.3)
Namibia	47.7% (45.8%, 49.5%)	40.1% (32.8%, 47.9%)	0.7 (0.5, 1.1)
Peru	58.6% (57.2%, 60.0%)	30.5% (24.8%, 36.0%)	0.3 (0.2, 0.4)
Philippines	43.5% (41.8%, 45.0%)	23.4% (17.4%, 30.1%)	0.4 (0.3, 0.6)
Pakistan	45.1% (43.8%, 46.3%)	29.3% (24.5%, 34.4%)	0.5 (0.4, 0.7)
Paraguay	39.5% (37.6%, 41.3%)	34.3% (27.0%, 41.9%)	0.8 (0.5, 1.2)
Rwanda	21.5% (20.7%, 22.4%)	10.5% (7.2%, 14.0%)	0.4 (0.3, 0.6)
Sierra Leone	32.0% (31.1%, 33.0%)	20.1% (16.3%, 24.0%)	0.5 (0.4, 0.7)
Senegal	33.0% (32.0%, 33.8%)	12.3% (8.8%, 16.2%)	0.3 (0.2, 0.4)
Sao Tome and Principe	40.3% (38.4%, 42.1%)	34.4% (27.0%, 42.1%)	0.8 (0.5, 1.2)
Eswatini	28.8% (26.8%, 30.6%)	31.0% (23.9%, 39.2%)	1.1 (0.7, 1.8)
Chad	20.5% (19.7%, 21.3%)	18.3% (15.2%, 21.5%)	0.9 (0.7, 1.1)
Togo	27.4% (26.4%, 28.4%)	14.3% (10.4%, 18.4%)	0.4 (0.3, 0.6)
Timor-Leste	25.1% (24.0%, 26.0%)	14.7% (11.0%, 18.8%)	0.5 (0.4, 0.7)
Turkey	71.8% (70.0%, 73.7%)	49.8% (42.4%, 57.0%)	0.4 (0.3, 0.6)
Tanzania	21.0% (19.7%, 22.3%)	24.0% (19.1%, 29.1%)	1.2 (0.8, 1.7)
Ukraine	54.3% (51.2%, 57.1%)	61.5% (50.5%, 73.8%)	1.4 (0.8, 2.7)
Uganda	20.0% (19.0%, 20.9%)	14.2% (10.7%, 18.1%)	0.7 (0.5, 0.9)
Uzbekistan	44.7% (42.4%, 47.1%)	42.9% (33.3%, 52.1%)	1.0 (0.6, 1.5)
Vietnam	18.6% (17.7%, 19.4%)	7.0% (3.9%, 10.8%)	0.3 (0.2, 0.6)
South Africa	51.7% (49.9%, 53.3%)	27.4% (20.9%, 34.5%)	0.4 (0.2, 0.5)
Zambia	38.1% (36.8%, 39.4%)	34.2% (29.1%, 39.4%)	0.9 (0.6, 1.1)
Zimbabwe	39.4% (37.8%, 40.9%)	23.4% (17.4%, 29.5%)	0.5 (0.3, 0.7)

Table 4 — Comparison of residence (proportion urban) between non-high risk and high risk births. Latest survey for each country.

Country	Low Risk	High Risk	Odds Ratio
Albania	6.4% (5.5%, 7.3%)	14.1% (10.5%, 17.7%)	2.5 (1.5, 3.7)
Armenia	3.1% (2.6%, 3.6%)	6.7% (4.9%, 8.7%)	2.3 (1.4, 3.6)
Angola	12.6% (11.0%, 14.3%)	58.4% (51.7%, 65.0%)	10.0 (6.4, 15.1)
Azerbaijan	8.8% (7.5%, 9.9%)	21.2% (16.6%, 26.1%)	2.9 (1.8, 4.3)
Bangladesh	12.8% (11.5%, 14.0%)	31.4% (26.8%, 36.5%)	3.2 (2.3, 4.4)
Burkina Faso	28.5% (27.2%, 29.7%)	65.2% (60.3%, 70.5%)	4.8 (3.6, 6.4)
Benin	6.7% (5.6%, 7.8%)	57.0% (52.6%, 61.4%)	18.8 (13.1, 26.7)
Bolivia	18.9% (17.3%, 20.3%)	50.5% (45.0%, 56.8%)	4.4 (3.2, 6.3)
Brazil	9.9% (8.7%, 11.1%)	37.5% (32.7%, 42.5%)	5.5 (3.9, 7.8)
Burundi	30.2% (28.5%, 32.0%)	47.1% (39.9%, 53.8%)	2.1 (1.4, 2.9)
Congo Democratic Republic	17.9% (16.5%, 19.2%)	65.9% (60.7%, 71.6%)	9.0 (6.5, 12.8)
Central African Republic	26.8% (25.0%, 28.8%)	44.4% (36.7%, 51.9%)	2.2 (1.4, 3.2)
Congo	17.6% (15.9%, 19.2%)	41.1% (34.7%, 47.9%)	3.3 (2.2, 4.9)
Côte d'Ivoire	19.9% (18.3%, 21.6%)	60.6% (54.0%, 67.1%)	6.3 (4.3, 9.1)
Cameroon	20.4% (19.0%, 21.8%)	59.1% (53.6%, 64.7%)	5.7 (4.1, 7.8)
Colombia	6.1% (5.4%, 6.7%)	12.6% (10.2%, 15.5%)	2.3 (1.6, 3.2)
Dominican Republic	7.3% (6.4%, 8.3%)	17.0% (13.3%, 20.9%)	2.6 (1.7, 3.9)
Egypt	4.1% (3.4%, 4.8%)	22.5% (19.6%, 25.5%)	6.9 (4.8, 9.8)
Ethiopia	27.0% (25.6%, 28.5%)	60.8% (55.0%, 66.3%)	4.3 (3.1, 5.7)
Gabon	11.3% (9.8%, 12.6%)	35.7% (30.2%, 41.5%)	4.5 (3.0, 6.5)
Ghana	18.6% (17.0%, 20.1%)	30.7% (24.6%, 37.2%)	2.0 (1.3, 2.9)
Guinea	23.6% (21.9%, 25.3%)	64.3% (57.4%, 71.2%)	6.0 (4.0, 8.8)
Guatemala	8.6% (7.4%, 9.8%)	28.5% (23.9%, 33.4%)	4.3 (2.9, 6.3)
Guyana	7.9% (6.8%, 8.9%)	15.7% (11.8%, 20.1%)	2.2 (1.4, 3.4)
Honduras	8.2% (7.3%, 9.0%)	15.8% (12.6%, 19.5%)	2.1 (1.4, 3.1)
Haiti	18.2% (16.5%, 19.9%)	42.6% (35.9%, 49.2%)	3.4 (2.3, 4.9)
India	9.9% (9.0%, 10.7%)	52.0% (48.9%, 55.4%)	9.9 (8.0, 12.5)
Indonesia	6.9% (6.1%, 7.8%)	33.8% (30.2%, 37.2%)	7.0 (5.1, 9.1)
Jordan	4.8% (3.9%, 5.7%)	20.5% (17.0%, 24.2%)	5.2 (3.4, 7.9)
Kenya	8.7% (7.6%, 9.9%)	44.8% (40.2%, 49.2%)	8.6 (6.2, 11.7)
Cambodia	8.3% (7.2%, 9.5%)	37.0% (32.2%, 41.6%)	6.6 (4.5, 9.2)
Kazakhstan	10.1% (8.7%, 11.3%)	18.1% (13.4%, 23.5%)	2.0 (1.2, 3.2)
Comoros	8.9% (7.5%, 10.2%)	29.4% (24.0%, 35.0%)	4.4 (2.8, 6.6)
Kyrgyzstan	5.8% (5.1%, 6.5%)	10.4% (7.6%, 13.5%)	1.9 (1.2, 2.9)
Liberia	28.9% (27.3%, 30.5%)	59.3% (52.8%, 65.6%)	3.6 (2.5, 5.1)
Lesotho	11.3% (9.9%, 12.6%)	23.8% (18.6%, 29.5%)	2.5 (1.6, 3.8)
Morocco	16.6% (14.8%, 18.1%)	38.7% (32.4%, 45.7%)	3.2 (2.2, 4.8)
Moldova	6.0% (5.5%, 6.4%)	5.0% (3.4%, 6.9%)	0.8 (0.5, 1.3)
Madagascar	13.4% (12.0%, 14.7%)	59.0% (53.6%, 64.6%)	9.5 (6.7, 13.5)
Mali	12.5% (11.0%, 13.9%)	69.3% (63.6%, 75.0%)	16.3 (10.9, 24.1)
Malawi	13.8% (12.3%, 15.2%)	53.9% (48.3%, 59.8%)	7.4 (5.2, 10.6)
Mozambique	18.3% (16.9%, 19.7%)	57.1% (51.7%, 62.9%)	6.0 (4.4, 8.3)
Nicaragua	16.0% (14.5%, 17.5%)	29.8% (23.5%, 35.8%)	2.3 (1.4, 3.3)
Nigeria	21.9% (21.1%, 22.7%)	81.0% (77.7%, 84.1%)	15.4 (11.9, 19.9)
Niger	29.6% (28.2%, 30.8%)	72.3% (67.5%, 77.8%)	6.3 (4.7, 8.9)
Namibia	6.9% (5.9%, 7.9%)	21.9% (18.0%, 26.0%)	3.9 (2.6, 5.6)
Peru	8.0% (6.9%, 9.0%)	27.8% (23.8%, 32.0%)	4.5 (3.2, 6.3)
Philippines	6.8% (6.0%, 7.6%)	15.6% (12.4%, 18.9%)	2.6 (1.7, 3.7)
Pakistan	13.0% (11.6%, 14.5%)	59.5% (53.6%, 65.2%)	10.0 (6.8, 14.3)
Paraguay	14.1% (12.3%, 15.7%)	29.8% (23.5%, 36.7%)	2.7 (1.7, 4.1)
Rwanda	26.6% (24.9%, 28.2%)	42.4% (36.0%, 49.4%)	2.1 (1.4, 2.9)
Sierra Leone	25.7% (24.5%, 26.8%)	76.4% (71.9%, 81.1%)	9.5 (7.0, 13.2)
Senegal	19.2% (17.5%, 20.9%)	49.1% (42.1%, 55.8%)	4.1 (2.7, 5.9)
Sao Tome and Principe	15.3% (13.9%, 16.6%)	22.7% (17.5%, 28.2%)	1.6 (1.1, 2.4)
Eswatini	11.6% (10.1%, 13.0%)	31.0% (25.4%, 37.1%)	3.5 (2.3, 5.3)
Chad	20.0% (18.9%, 21.2%)	75.7% (71.1%, 80.2%)	12.7 (9.2, 17.4)
Togo	18.6% (16.8%, 20.3%)	49.3% (42.6%, 56.5%)	4.3 (2.9, 6.4)
Timor-Leste	20.4% (18.8%, 22.1%)	56.3% (49.6%, 62.8%)	5.1 (3.5, 7.3)
Turkey	12.0% (10.5%, 13.6%)	38.3% (31.8%, 44.3%)	4.7 (3.0, 6.8)
Tanzania	18.5% (16.8%, 20.2%)	44.4% (37.7%, 51.3%)	3.6 (2.4, 5.2)
Ukraine	2.3% (2.2%, 2.4%)	0.7% (0.3%, 1.3%)	0.3 (0.1, 0.6)
Uganda	29.5% (27.8%, 31.1%)	46.4% (39.8%, 53.2%)	2.1 (1.5, 3.0)
Uzbekistan	8.0% (6.7%, 9.3%)	30.1% (24.8%, 35.3%)	5.1 (3.2, 7.6)
Vietnam	10.0% (8.8%, 11.2%)	14.9% (10.3%, 19.8%)	1.6 (0.9, 2.6)
South Africa	9.9% (8.6%, 11.2%)	28.9% (23.7%, 34.1%)	3.8 (2.5, 5.5)
Zambia	19.8% (18.4%, 21.2%)	48.8% (43.5%, 54.4%)	3.9 (2.9, 5.3)
Zimbabwe	8.1% (6.9%, 9.3%)	30.7% (25.9%, 35.5%)	5.1 (3.4, 7.4)

Table 5 — Comparison of prior death of a child between non-high risk and high risk births. Latest survey for each country.

Country	Low Risk	High Risk	Difference
Albania	0.52 (0.51, 0.53)	0.38 (0.33, 0.43)	-0.14 (-0.2, -0.08)
Armenia	0.52 (0.5, 0.54)	0.51 (0.44, 0.58)	-0.02 (-0.11, 0.07)
Angola	0.51 (0.51, 0.52)	0.43 (0.39, 0.46)	-0.09 (-0.13, -0.05)
Azerbaijan	0.48 (0.47, 0.5)	0.42 (0.37, 0.47)	-0.06 (-0.13, 0)
Bangladesh	0.52 (0.51, 0.53)	0.36 (0.32, 0.39)	-0.16 (-0.2, -0.12)
Burkina Faso	0.52 (0.51, 0.52)	0.41 (0.38, 0.43)	-0.11 (-0.14, -0.07)
Benin	0.51 (0.5, 0.51)	0.41 (0.39, 0.44)	-0.09 (-0.12, -0.07)
Bolivia	0.52 (0.52, 0.53)	0.32 (0.3, 0.35)	-0.2 (-0.24, -0.16)
Brazil	0.53 (0.52, 0.54)	0.31 (0.28, 0.35)	-0.22 (-0.27, -0.17)
Burundi	0.53 (0.52, 0.54)	0.39 (0.35, 0.42)	-0.14 (-0.18, -0.09)
Congo Democratic Republic	0.51 (0.5, 0.51)	0.42 (0.4, 0.45)	-0.08 (-0.12, -0.05)
Central African Republic	0.52 (0.51, 0.53)	0.41 (0.37, 0.44)	-0.11 (-0.16, -0.07)
Congo	0.51 (0.5, 0.52)	0.44 (0.41, 0.48)	-0.07 (-0.11, -0.02)
Côte d'Ivoire	0.5 (0.5, 0.51)	0.46 (0.43, 0.49)	-0.04 (-0.08, -0.01)
Cameroon	0.53 (0.52, 0.53)	0.32 (0.29, 0.34)	-0.21 (-0.24, -0.18)
Colombia	0.52 (0.51, 0.53)	0.35 (0.31, 0.39)	-0.18 (-0.23, -0.12)
Dominican Republic	0.5 (0.49, 0.51)	0.38 (0.33, 0.43)	-0.12 (-0.18, -0.06)
Egypt	0.55 (0.54, 0.56)	0.39 (0.36, 0.43)	-0.16 (-0.2, -0.11)
Ethiopia	0.51 (0.5, 0.51)	0.42 (0.39, 0.45)	-0.09 (-0.12, -0.05)
Gabon	0.52 (0.51, 0.53)	0.41 (0.37, 0.45)	-0.11 (-0.16, -0.06)
Ghana	0.52 (0.51, 0.53)	0.41 (0.37, 0.45)	-0.11 (-0.16, -0.05)
Guinea	0.52 (0.51, 0.53)	0.38 (0.35, 0.41)	-0.14 (-0.18, -0.1)
Guatemala	0.51 (0.5, 0.52)	0.36 (0.32, 0.39)	-0.16 (-0.2, -0.11)
Guyana	0.45 (0.43, 0.46)	0.52 (0.47, 0.58)	0.07 (0.01, 0.15)
Honduras	0.51 (0.5, 0.52)	0.39 (0.35, 0.44)	-0.11 (-0.17, -0.06)
Haiti	0.5 (0.49, 0.51)	0.46 (0.42, 0.5)	-0.04 (-0.08, 0.01)
India	0.52 (0.52, 0.53)	0.26 (0.25, 0.27)	-0.26 (-0.28, -0.25)
Indonesia	0.54 (0.53, 0.55)	0.28 (0.25, 0.3)	-0.26 (-0.29, -0.23)
Jordan	0.52 (0.51, 0.53)	0.45 (0.4, 0.49)	-0.07 (-0.13, -0.02)
Kenya	0.49 (0.48, 0.49)	0.46 (0.43, 0.48)	-0.03 (-0.06, 0)
Cambodia	0.53 (0.52, 0.54)	0.32 (0.28, 0.35)	-0.21 (-0.25, -0.18)
Kazakhstan	0.49 (0.48, 0.5)	0.39 (0.33, 0.45)	-0.1 (-0.17, -0.03)
Comoros	0.49 (0.48, 0.5)	0.48 (0.43, 0.52)	-0.01 (-0.06, 0.04)
Kyrgyzstan	0.51 (0.49, 0.52)	0.52 (0.46, 0.57)	0.01 (-0.06, 0.08)
Liberia	0.51 (0.5, 0.52)	0.45 (0.42, 0.48)	-0.06 (-0.1, -0.02)
Lesotho	0.47 (0.46, 0.48)	0.5 (0.46, 0.55)	0.03 (-0.02, 0.09)
Morocco	0.52 (0.51, 0.53)	0.34 (0.3, 0.38)	-0.18 (-0.22, -0.13)
Moldova	0.5 (0.48, 0.51)	0.46 (0.41, 0.51)	-0.04 (-0.1, 0.03)
Madagascar	0.52 (0.51, 0.52)	0.37 (0.35, 0.4)	-0.14 (-0.17, -0.11)
Mali	0.51 (0.5, 0.51)	0.43 (0.4, 0.45)	-0.08 (-0.11, -0.05)
Malawi	0.51 (0.51, 0.52)	0.41 (0.38, 0.44)	-0.11 (-0.14, -0.07)
Mozambique	0.5 (0.5, 0.51)	0.41 (0.38, 0.44)	-0.09 (-0.13, -0.05)
Nicaragua	0.5 (0.5, 0.51)	0.36 (0.33, 0.4)	-0.14 (-0.18, -0.1)
Nigeria	0.53 (0.53, 0.54)	0.32 (0.31, 0.33)	-0.21 (-0.23, -0.2)
Niger	0.5 (0.49, 0.5)	0.43 (0.41, 0.46)	-0.06 (-0.09, -0.03)
Namibia	0.52 (0.51, 0.53)	0.42 (0.38, 0.46)	-0.1 (-0.15, -0.05)
Peru	0.53 (0.53, 0.54)	0.3 (0.27, 0.32)	-0.24 (-0.27, -0.2)
Philippines	0.52 (0.51, 0.53)	0.34 (0.3, 0.38)	-0.18 (-0.23, -0.13)
Pakistan	0.5 (0.49, 0.5)	0.36 (0.33, 0.39)	-0.14 (-0.17, -0.1)
Paraguay	0.53 (0.52, 0.54)	0.42 (0.38, 0.47)	-0.11 (-0.16, -0.05)
Rwanda	0.51 (0.5, 0.52)	0.4 (0.36, 0.43)	-0.11 (-0.16, -0.07)
Sierra Leone	0.5 (0.5, 0.51)	0.42 (0.39, 0.44)	-0.09 (-0.12, -0.05)
Senegal	0.49 (0.48, 0.5)	0.31 (0.27, 0.34)	-0.19 (-0.23, -0.14)
Sao Tome and Principe	0.5 (0.49, 0.51)	0.49 (0.44, 0.53)	-0.01 (-0.07, 0.05)
Eswatini	0.52 (0.51, 0.53)	0.5 (0.46, 0.55)	-0.02 (-0.08, 0.04)
Chad	0.51 (0.5, 0.51)	0.45 (0.43, 0.48)	-0.06 (-0.08, -0.03)
Togo	0.52 (0.51, 0.53)	0.4 (0.37, 0.43)	-0.12 (-0.16, -0.08)
Timor-Leste	0.52 (0.51, 0.53)	0.44 (0.41, 0.47)	-0.08 (-0.12, -0.04)
Turkey	0.52 (0.51, 0.53)	0.3 (0.27, 0.34)	-0.22 (-0.26, -0.17)
Tanzania	0.49 (0.48, 0.5)	0.46 (0.42, 0.5)	-0.03 (-0.07, 0.02)
Ukraine	0.49 (0.47, 0.5)	0.52 (0.45, 0.59)	0.04 (-0.05, 0.13)
Uganda	0.5 (0.49, 0.51)	0.4 (0.37, 0.44)	-0.1 (-0.14, -0.05)
Uzbekistan	0.48 (0.47, 0.49)	0.47 (0.42, 0.52)	-0.01 (-0.07, 0.06)
Vietnam	0.5 (0.49, 0.51)	0.36 (0.32, 0.4)	-0.14 (-0.19, -0.08)
South Africa	0.52 (0.51, 0.53)	0.31 (0.28, 0.35)	-0.21 (-0.25, -0.16)
Zambia	0.51 (0.5, 0.52)	0.41 (0.38, 0.44)	-0.1 (-0.14, -0.06)
Zimbabwe	0.52 (0.51, 0.52)	0.4 (0.36, 0.44)	-0.12 (-0.16, -0.07)

Table 6 — Comparison of wealth CDF between non-high risk and high risk births. Latest survey for each country.

Country	Low Risk	High Risk	Difference
Albania	2.37 (2.32, 2.42)	2.59 (2.39, 2.79)	0.21 (-0.03, 0.46)
Armenia	1.8 (1.75, 1.85)	1.94 (1.76, 2.13)	0.15 (-0.07, 0.38)
Angola	3.12 (3.06, 3.2)	3.76 (3.46, 4.03)	0.64 (0.28, 0.97)
Azerbaijan	2.14 (2.1, 2.19)	2.3 (2.11, 2.49)	0.16 (-0.09, 0.39)
Bangladesh	2.4 (2.35, 2.46)	3.12 (2.9, 3.36)	0.72 (0.44, 1.03)
Burkina Faso	3.63 (3.57, 3.69)	4.26 (4.01, 4.52)	0.63 (0.31, 0.95)
Benin	3.07 (3.02, 3.12)	4.32 (4.12, 4.53)	1.25 (1, 1.51)
Bolivia	3.22 (3.15, 3.3)	4.57 (4.26, 4.87)	1.35 (0.96, 1.72)
Brazil	2.72 (2.65, 2.79)	3.9 (3.62, 4.17)	1.18 (0.83, 1.53)
Burundi	3.7 (3.62, 3.79)	3.61 (3.27, 3.95)	-0.1 (-0.52, 0.33)
Congo Democratic Republic	3.57 (3.5, 3.63)	4.47 (4.23, 4.73)	0.9 (0.58, 1.23)
Central African Republic	3.7 (3.61, 3.78)	3.58 (3.25, 3.93)	-0.12 (-0.5, 0.32)
Congo	3.19 (3.11, 3.26)	3.88 (3.59, 4.18)	0.69 (0.31, 1.07)
Côte d'Ivoire	3.37 (3.29, 3.45)	4.37 (4.05, 4.68)	1 (0.6, 1.39)
Cameroon	3.46 (3.39, 3.52)	4.39 (4.14, 4.66)	0.93 (0.6, 1.26)
Colombia	2.46 (2.4, 2.51)	3.06 (2.83, 3.28)	0.6 (0.33, 0.87)
Dominican Republic	2.53 (2.46, 2.59)	2.79 (2.53, 3.05)	0.26 (-0.06, 0.59)
Egypt	2.41 (2.36, 2.46)	2.81 (2.61, 2.99)	0.4 (0.16, 0.62)
Ethiopia	3.76 (3.69, 3.82)	4.4 (4.14, 4.69)	0.65 (0.31, 1)
Gabon	3.47 (3.38, 3.56)	4.3 (3.95, 4.66)	0.83 (0.4, 1.28)
Ghana	3.23 (3.16, 3.3)	3.59 (3.29, 3.88)	0.36 (0, 0.73)
Guinea	3.54 (3.46, 3.62)	4.45 (4.15, 4.78)	0.91 (0.54, 1.32)
Guatemala	3 (2.92, 3.09)	4.12 (3.78, 4.46)	1.12 (0.7, 1.54)
Guyana	3.21 (3.12, 3.29)	3.03 (2.72, 3.38)	-0.18 (-0.58, 0.25)
Honduras	3.05 (2.97, 3.13)	3.25 (2.95, 3.58)	0.2 (-0.18, 0.61)
Haiti	3.26 (3.17, 3.34)	4.24 (3.91, 4.59)	0.99 (0.56, 1.43)
India	2.58 (2.55, 2.61)	3.72 (3.59, 3.85)	1.13 (0.98, 1.3)
Indonesia	2.39 (2.35, 2.43)	3.2 (3.03, 3.36)	0.8 (0.59, 1.01)
Jordan	3.37 (3.29, 3.45)	3.87 (3.55, 4.19)	0.51 (0.1, 0.94)
Kenya	3.26 (3.2, 3.31)	3.94 (3.71, 4.16)	0.68 (0.4, 0.96)
Cambodia	2.48 (2.42, 2.54)	3.74 (3.5, 3.98)	1.26 (0.95, 1.55)
Kazakhstan	2.3 (2.24, 2.36)	2.37 (2.14, 2.63)	0.07 (-0.23, 0.38)
Comoros	3.46 (3.37, 3.55)	4.04 (3.69, 4.4)	0.57 (0.15, 1.02)
Kyrgyzstan	2.55 (2.49, 2.6)	2.31 (2.08, 2.53)	-0.24 (-0.53, 0.04)
Liberia	3.65 (3.57, 3.73)	4.42 (4.08, 4.75)	0.78 (0.35, 1.19)
Lesotho	2.59 (2.52, 2.64)	2.64 (2.4, 2.89)	0.06 (-0.23, 0.36)
Morocco	3.4 (3.31, 3.48)	3.89 (3.55, 4.24)	0.49 (0.06, 0.93)
Moldova	1.9 (1.87, 1.93)	1.82 (1.69, 1.96)	-0.08 (-0.24, 0.09)
Madagascar	3.4 (3.33, 3.47)	4.5 (4.22, 4.77)	1.1 (0.75, 1.46)
Mali	3.26 (3.19, 3.32)	4.41 (4.15, 4.69)	1.15 (0.81, 1.5)
Malawi	3.2 (3.15, 3.27)	3.96 (3.72, 4.2)	0.76 (0.44, 1.06)
Mozambique	3.22 (3.16, 3.28)	3.43 (3.19, 3.67)	0.21 (-0.08, 0.53)
Nicaragua	3.6 (3.5, 3.7)	4.27 (3.88, 4.66)	0.67 (0.19, 1.16)
Nigeria	3.61 (3.56, 3.65)	5.12 (4.94, 5.3)	1.51 (1.29, 1.73)
Niger	3.99 (3.92, 4.06)	4.38 (4.11, 4.67)	0.39 (0.05, 0.75)
Namibia	2.72 (2.65, 2.78)	2.86 (2.59, 3.11)	0.14 (-0.17, 0.46)
Peru	2.66 (2.6, 2.72)	3.73 (3.51, 3.96)	1.07 (0.81, 1.36)
Philippines	2.92 (2.84, 3)	3.77 (3.46, 4.09)	0.85 (0.45, 1.26)
Pakistan	3.49 (3.42, 3.57)	4.66 (4.37, 4.95)	1.16 (0.8, 1.53)
Paraguay	3.79 (3.67, 3.9)	4.39 (3.94, 4.85)	0.6 (0.03, 1.18)
Rwanda	3.1 (3.02, 3.17)	3.46 (3.15, 3.76)	0.36 (-0.02, 0.74)
Sierra Leone	3.3 (3.24, 3.36)	4.67 (4.42, 4.9)	1.37 (1.06, 1.67)
Senegal	3.48 (3.4, 3.56)	4.17 (3.84, 4.5)	0.69 (0.27, 1.1)
Sao Tome and Principe	3.23 (3.14, 3.31)	3.67 (3.34, 4.04)	0.45 (0.04, 0.92)
Eswatini	3.24 (3.15, 3.33)	3.7 (3.34, 4.05)	0.46 (0.01, 0.9)
Chad	4 (3.95, 4.06)	4.91 (4.68, 5.12)	0.9 (0.62, 1.18)
Togo	3.39 (3.31, 3.46)	4.13 (3.83, 4.42)	0.74 (0.37, 1.1)
Timor-Leste	3.9 (3.82, 3.98)	4.21 (3.88, 4.53)	0.32 (-0.1, 0.71)
Turkey	2.73 (2.65, 2.81)	3.97 (3.63, 4.29)	1.23 (0.81, 1.64)
Tanzania	3.6 (3.51, 3.69)	4.26 (3.91, 4.62)	0.66 (0.22, 1.11)
Ukraine	1.58 (1.54, 1.61)	1.66 (1.52, 1.8)	0.09 (-0.09, 0.27)
Uganda	4.02 (3.93, 4.11)	4.21 (3.86, 4.57)	0.2 (-0.24, 0.66)
Uzbekistan	2.93 (2.84, 3)	2.94 (2.63, 3.26)	0.02 (-0.37, 0.43)
Vietnam	2.44 (2.36, 2.5)	2.71 (2.44, 3)	0.27 (-0.07, 0.63)
South Africa	2.72 (2.65, 2.78)	3.36 (3.1, 3.64)	0.64 (0.31, 1)
Zambia	3.49 (3.43, 3.56)	4.39 (4.11, 4.65)	0.91 (0.56, 1.23)
Zimbabwe	2.49 (2.43, 2.54)	3.12 (2.9, 3.35)	0.63 (0.35, 0.92)

Table 7 — Comparison of birth order between non-high risk and high risk births. Latest survey for each country.
