# Supplementary material for: Leave no child behind: Using data from 1.7 million children from 67 developing countries to measure inequality within and between groups of births and to identify left behind populations
Source: PLoS One. 2020 Oct 14;15(10):e0238847. doi: 10.1371/journal.pone.0238847 (PMC7556530; doi:10.1371/journal.pone.0238847)
Supplement: Attachment — Submitted filename: PONE-D-19-25716_R1_review_20200206.pdf [file pone.0238847.s003.pdf]

The authors provide new insight into ways of describing and reporting inequalities in U5MR. The authors also introduce new metrics into understanding associations between independent variables and U5MR such as the prior death variable and the method of standardizing DHS wealth indices across surveys, which is a recurrent problem for researchers using DHS data for cross-national comparisons. The authors first revision improved the paper greatly. I only have a few remaining comments and some additional copy-edits.

1 Broad comments

- At the bottom of pg. 9 through the top of pg. 10 the authors write, "Because wealth information is collected at the household level, and poorer households usually have have more births than richer household, among births the median average wealth will not necessarily be 50%." I do not understand the sentiment the sentence is trying to convey. Do you the median wealth amongst births will be lower in value than amongst households? The sentence is unclear.

- In section 3.1, the authors state "The majority of deaths are not from the poorest 20% of families." Do you mean the poorest 20% of births? It may create less confusion for the reader if you refer to all percentiles in terms of either births or families.

- Bayesian ANOVA: It appears from Appendix A3 that you perform 1000 tests for each survey, but you only report one R^2 and one p-value in Table 2. To address multiple testing issues, a Bonferroni correction would require a threshhold of 0.05/1000 or 0.00005 to be significant. So reporting the p-value as ¡0.0001 is not necessarily scientifically important if the authors wish to use a p-value cutoff. This still does not address why there is only one R^2 and one p-value reported. I would recommend the authors report the 95% interval from the distribution of R^2. This would make the results slightly more descriptive than formal hypothesis testing, but would circumnavigate the multiple testing issue. Alternatively, the authors might report the intervals for both R^2 and the p-values. In either case, one of the benefits of the Bayesian analysis here is to capture uncertainty across parameters and functions of parameters in the model, and it would be nice to see that benefit in the results.

- Following the previous comment, is there a reason the authors choose to report uncertainty in certain measures and not others. For example, in Table 3, there is uncertainty reported for the highest risk group, but not for the poorest.

- Addressing the complex survey design: DHS surveys tend to be stratified two-stage cluster sampling. Strata tend to be urban/rural crossed with Admin-1 or some geographic division. Not accounting for strata from the survey design can lead to bias in estimators and not accounting for clustering can lead to improper variance estimates. While the model inherently does this by including urban/rural as a covariate, and the authors address the first stage dependence amongst clusters, it would be great to see the complex survey design and how it is addressed in modeling

explicitly mentioned in the text. To that end, the descriptive statistics in Table 1 contain mean estimates of U5MR and mean estimates by quintile. Are survey weights accounted for in these estimates or are they straight sample means as if the survey design were a simple random sample? DHS may oversample certain strata so it is important that the sampling weights are used here to get estimates.

- Geographic location of clusters: The authors mention they use geographic location, but actually that is not available for all DHS and these locations, when available are jittered. The introduction to including this variable is misleading as what the authors actually include is cluster or primary sampling unit (PSU) index. While each cluster index is unique to a location, there is no spatial information actually being incorporated in your model, only addressing of dependence between children in the same PSU. (which is very important!)

2 Small Comments

- In the abstract and introduction there are multiple places where the SDG acronym is introduced. Additionally, it is once introduced as SDG and another time introduced as SDGs.
- In the Introduction the authors write they use a "...Bayesian hierarchical model to estimate mortality...". I would be specific here and state that you are estimating the probability or risk of mortality.
- In Section 2.2 the authors write they include two other variables, but it appears they add three: clusters, previous death, and birth order.
- At the end of Section 2.2 the authors write "We then identify hte 20% high risk", perhaps replace "high" with "highest".
- In Section 2.3, the authors write they multiple the efficiency gain metric "times 100%." This should say "times 100."
- Be consistent in how you refer to urban/rural it is sometimes "urban rural" and some times "urban rural" and sometimes "urban-rural" in the text.
- In Section 3.2 replace all references to "pvalue" with "p-value".
- In Section 3.2 the authors state: "...and pvalues shown whether..." replace "shown" with "show".
- In Section 3.2 capitalize the references figure 2 and table 1.
- In Section 3.4 there is some confusing diction around the percentiles. I would write the "22nd percentile" and the "53rd percentile". In the following sentence remove the word "percentile" from the results for Peru.

- In the Discussion the authors write “distributions... are right skewed, because births that experience...”. I think this should be worded differently, perhaps: “right skewed, because some births experience...”
- In the Discussion the authors write “information to indentify high risks births.”, replace “risks” with “risk”.
- In the Discussion the authors write “Also, every comparison allocation scheme also needs to accommodate...”. Remove one of “also” or “Also”.
- In Appendix A1 the authors write “exactly 20% of each birth” perhaps it would read better as “It does not have exactly 20% of births in each quintile...”
- In Appendix A3 The second sentence begins “Variance Var(Π)”, remove “Variance”.
