# Supplementary material for: Leave no child behind: Using data from 1.7 million children from 67 developing countries to measure inequality within and between groups of births and to identify left behind populations
Source: PLoS One. 2020 Oct 14;15(10):e0238847. doi: 10.1371/journal.pone.0238847 (PMC7556530; doi:10.1371/journal.pone.0238847)
Supplement: Attachment — Submitted filename: response_to_reviewers_PlosOne _2.docx [file pone.0238847.s004.docx]

The authors provide new insight into ways of describing and reporting inequalities in U5MR. The authors also introduce new metrics into understanding associations between independent variables and U5MR such as the prior death variable and the method of standardizing DHS wealth indices across surveys, which is a recurrent problem for researchers using DHS data for cross-national comparisons. The authors first revision improved the paper greatly. I only have a few remaining comments and some additional copy-edits.

Thank you for the kind comments and for your help.

1 Broad comments

• At the bottom of pg. 9 through the top of pg. 10 the authors write, “Because wealth information is collected at the household level, and poorer households usually have more births than richer household, among births the median average wealth will not necessarily be 50%.” I do not understand the sentiment the sentence is trying to convey. Do you the median wealth amongst births will be lower in value than amongst households? The sentence is unclear.

Thank you for letting us clarify this important point. (a) Wealth information is collected at the household level, not at the birth level; (c) therefore all births from the same household have the same wealth level; (b) Poorer households usually have higher fertility than richer households; (c) Thus median wealth among births is not the same as median wealth among households, rather median wealth for births is (usually) lower than median wealth for households.

Suppose there are 101 households, ranked from richest to poorest. Wealth data is collected at the household level and all households have 1 birth each. In that case, the birth with median wealth was born to the 51st household, which is also the median wealth household. Now suppose that fertility is higher among poorer households such that the 10 poorest households have 2 births each but all other households have just one birth. Now the median wealth birth was born to the 56th ranked household.

To clarify this point we have added material to appendix A2 and we have changed the original sentence in the main text to:

"Because information on wealth is collected at the household level, all births from the same household have the same wealth level. Thus we re-score birth-wealth to be on a 0-1 scale so that on the birth-wealth ranking, the median is.5. The re-scored median birth is born to a household that is below the median household wealth because poorer households usually have higher fertility than richer households. Please see details in the appendix."

• In section 3.1, the authors state “The majority of deaths are not from the poorest 20% of families.” Do you mean the poorest 20% of births? It may create less confusion for the reader if you refer to all percentiles in terms of either births or families.

Thank you for identifying this error in our text. Since our analysis is at the individual birth level, we intended to talk about wealth ranks for births. We will make changes in the text accordingly. We have changed that sentence to “The majority of deaths are not from the poorest 20% of births.”

• Bayesian ANOVA: It appears from Appendix A3 that you perform 1000 tests for each survey, but you only report one R^2^ and one p-value in Table 2. To address multiple testing issues, a Bonferroni correction would require a threshold of 0.05/1000 or 0.00005 to be significant. So reporting the p-value as <.0001 is not necessarily scientifically important if the authors wish to use a p-value cutoff. This still does not address why there is only one R^2^ and one p-value reported. I would recommend the authors report the 95% interval from the distribution of R^2^. This would make the results slightly more descriptive than formal hypothesis testing, but would circumnavigate the multiple testing issue. Alternatively, the authors might report the intervals for both R^2^ and the p-values. In either case, one of the benefits of the Bayesian analysis here is to capture uncertainty across parameters and functions of parameters in the model, and it would be nice to see that benefit in the results.

Thank you for your comments. We agree on your take on the benefits of the Bayesian analysis. The 1000 R^2 calculations are not independent and the 1000 associated p-values are not independent; rather the 1000 R^2 and the 1000 p-values represent 1000 posterior samples from the posterior of R^2 and from the posterior of the p-value respectively. That is, there is only one R^2 and one p-value (and thus no need to make a Bonferroni correction), but because of uncertainty in the underlying true probabilities (mortality risks) we do not know the exact value of R^2 nor the exact value of the p-value. Previously we reported the posterior mean of R^2 and the posterior mean of the p-value. We have changed table 2 and the text and now we report a point estimated (expected values) and a 95% credible interval for the distributions of R^2 across our 1000 MCMC samples.

• Following the previous comment, is there a reason the authors choose to report uncertainty in certain measures and not others. For example, in Table 3, there is uncertainty reported for the highest risk group, but not for the poorest.

Thank you for your question. We do not report uncertainty for mortality among the poor on Table 3 because we calculated this quantity based on the raw data. It is a summary of the raw data, the proportion of deaths in the sample. There is no estimation involved. In general, we report uncertainty when our calculations come from statistical analysis, and not when they summarize the raw data. We take a Bayesian viewpoint: data is fixed once observed, but parameters (and functions of the parameters like R^2) are unknown and have uncertainty attached to them.

• Addressing the complex survey design: DHS surveys tend to be stratified two-stage cluster sampling. Strata tend to be urban/rural crossed with Admin-1 or some geographic division. Not accounting for strata from the survey design can lead to bias in estimators and not accounting for clustering can lead to improper variance estimates. While the model inherently does this by including urban/rural as a covariate, and the authors address the first stage dependence amongst clusters, it would be great to see the complex survey design and how it is addressed in modeling explicitly mentioned in the text. To that end, the descriptive statistics in Table 1 contain mean estimates of U5MR and mean estimates by quintile. Are survey weights accounted for in these estimates or are they straight sample means as if the survey design were a simple random sample? DHS may oversample certain strata so it is important that the sampling weights are used here to get estimates.

We have not included the complex sampling design from the DHS for simplicity with regard to our already complex methodological innovations. We did the modeling as well as we could, but did not reweight by the weights due to additional complexity that would introduce into our analysis but we now plan to look into this for future research. We are treating the given sample as a random sample when we estimate the density or CDF of the pi_i's. However, we have included all variables used to stratify the survey. We acknowledge this limitation in our discussion section of the new version of the paper. We suggest that in the future, researchers should incorporate complex surveys designs more explicitly in their analysis.

• Geographic location of clusters: The authors mention they use geographic location, but actually that is not available for all DHS and these locations, when available are jittered. The introduction to including this variable is misleading as what the authors actually include is cluster or primary sampling unit (PSU) index. While each cluster index is unique to a location, there is no spatial information actually being incorporated in your model, only addressing of dependence between children in the same PSU. (which is very important!)

Yes, this is correct. Thus we are not using geospatial location and a spatial model in our analysis. Still, we did model the dependence of children from the same cluster in our analysis, which is why we include sampling clusters as a random effect in our analysis.

2 Small Comments

• In the abstract and introduction there are multiple places where the SDG acronym is introduced. Additionally, it is once introduced as SDG and another time introduced as SDGs.

Thank you. We have fixed this in the latest version of the paper.

• In the Introduction the authors write they use a “...Bayesian hierarchical model to estimate mortality...”. I would be specific here and state that you are estimating the probability or risk of mortality.

Thank you. We have changed this.

• In Section 2.2 the authors write they include two other variables, but it appears they add three: clusters, previous death, and birth order.

Yes, we included three additional variables. Thank you for pointing this out. This is now fixed in the latest version of the paper.

• At the end of Section 2.2 the authors write “We then identify hte 20% high risk”, perhaps replace “high” with “highest”.

Thank you. The misspelling has been fixed.

• In Section 2.3, the authors write they multiple the efficiency gain metric “times 100%.” This should say “times 100.”

Thank you. Changed.

• Be consistent in how you refer to urban/rural it is sometimes “urban rural” and some times “urban rural” and sometimes “urban-rural” in the text.

Thank you. It is now consistent everywhere.

• In Section 3.2 replace all references to “pvalue” with “p-value”.

OK. Thank you. Done.

• In Section 3.2 the authors state: “...and pvalues shown whether...” replace “shown” with “show”.

Done.

• In Section 3.2 capitalize the references figure 2 and table 1.

Done.

• In Section 3.4 there is some confusing diction around the percentiles. I would write the “22^nd^ percentile” and the “53^rd^ percentile”. In the following sentence remove the word “percentile” from the results for Peru.

Done.

• In the Discussion the authors write “distributions... are right skewed, because births that experience...”. I think this should be worded differently, perhaps: “right skewed, because some births experience...”

We have now written “distribution … are right skewed, because some births experience substantially more mortality risk than the national average”.

• In the Discussion the authors write “information to identify high risks births.”, replace “risks” with “risk”.

Done.

• In the Discussion the authors write “Also, every comparison allocation scheme also needs to accommodate...”. Remove one of “also” or “Also”.

Done.

• In Appendix A1 the authors write “exactly 20% of each birth” perhaps it would read better as “It does not have exactly 20% of births in each quintile...”

Done.

• In Appendix A3 The second sentence begins “Variance Var(Π)”, remove “Variance”.

Done.

Editor Comments

- You do not seem to account for age of the child in the model, and as a result, the estimates presented in Table 1 should not be interpreted as under-five mortality rates, these are not comparable to estimates reported in DHS reports or UN IGME values (which are period 5Q0). Your estimates, if I understand correctly the methodology, are estimates referring to a cohort of children born between 5 and 10 years before the survey. This should be highlighted in the manuscript (on page 10?). Also note that Table 1 uses CMR for under-five mortality, which is a bit confusing since it is often used for child mortality, from age 1 to 5. Could you change to U5MR (from birth to age 5)? There is no need to specify under-five mortality rates "by age 5", since by definition U5MR is the risk of a newborn dying before age 5.

Thank you for highlighting these points. Table 1 is simply the proportion of deaths below the age of 5 for children born between 5-10 years before the survey. There are no modelling assumptions involved. We have now highlighted it in our manuscript on pp. 10. Similarly, we now use U5MR.

- The fact that U5MR sometimes increases from the lowest to the second quintile is also observed with estimates from DHS, but this increase is not necessarily significant. Would it be possible to add confidence intervals in Table 1?

Table 1 only reports raw data summaries – uncertainty estimates for example by treating the data as iid would not really be appropriate. Rather, we report uncertainty measures as output from the results of our statistical modeling.

- You indicate that you transformed the original wealth index but refer the readers to the appendix. Could you add one sentence in the main text about this transformation to explain how your index differs from the standard DHS index?

Yes, we have included a new sentence in the main text to clarify this point: " Because information on wealth is collected at the household level, all births from the same household have the same wealth level. Thus we re-score birth-wealth to be on a 0-1 scale so that on the birth-wealth ranking, the median is around .5. The re-scored median birth is born to a household that is below the median household wealth because poorer households usually have higher fertility than richer households. Please see details in the appendix."

- Figure 1 presents box plots showing the distribution of mortality risk. To what unit of observation do these risks correspond? To each child? Or is this a distribution of predicted proportions of children deceased before age five for each combination of the covariates? This is unclear. You state that variability is correlated with median mortality levels. How is the variability estimated here? On an absolute scale? In that case, it is logical that the variability is greater in high mortality countries. Please explain.

The data plot in the box plots correspond to mortality risk estimated for individual births. They are mortality risks for each birth based on their observed covariates, variability is assessed across all births or across all births in a defined subset (such as quintiles of wealth) of births. We present variability visually using box plots as well as taking the variance of the risks across all births that are present on table.

We have the following sentence as the first sentence in the results section of the paper: “All results use births (mortality risks) as the unit of analysis.”

A few typos:

1) in the abstract (interpretation), differences do not explain

2) In the caption of Table 1, fifth quantile instead of firth quantile, and I suggest mentioning "births from the richest households" and "births from the poorest households" instead of referring to rich and poor births.

Done

3) Brurundi on page 27

Done

Some minor comments:

1) on page 7, you indicate that "We analyze under-5 mortality and thus we exclude births that did not occur at least five years prior to the survey." Under-five mortality is sometimes estimated for periods up to the survey, by assuming that mortality rates of the older age groups are kept constant or decline at a certain rate, hence suggest removing "thus" in this sentence.

Done.
